# Unusual Case of a Missing Vibrator Device in the Pelvis

**DOI:** 10.1155/2020/8023798

**Published:** 2020-02-21

**Authors:** Greg J. Marchand, Katelyn M. Sainz, Ali Azadi, Alexa King, Sienna Anderson, Stacy Ruther, Giovanna Brazil, Lisa Rials, Kelly Ware, Asya Osborn, Sophia Hopewell

**Affiliations:** ^1^The Marchand Institute for Minimally Invasive Surgery, Mesa, AZ 85209, USA; ^2^Washington University of Health and Science, San Pedro, Belize; ^3^International University of the Health Sciences, St. Kitts, USA

## Abstract

Emergency room admissions and surgery secondary to the malfunctioning of devices intended for sexual stimulation are extremely common. Emergency room staff in the United States are commonly skilled in the detection and removal of some of these frequent occurrences. Occasionally, surgical intervention can be warranted if the device enters a cavity that cannot safely be explored in the emergency room setting. We report a case of a vibrator which was lost during sexual activity and appeared on flat plate X-ray to be in the abdominal cavity. A careful history showed that the device was of an unusually narrow diameter, and surgical intervention showed the device ultimately ended up in the bladder without traumatic injury. Following laparoscopic confirmation of the device's location in the bladder, cystoscopic removal was performed and the patient recovered uneventfully.

## 1. Introduction

Injuries related to sexual activity and usage of devices related to sexual activity are a very common cause of emergency room presentation [1]. Malfunction of these items (particularly devices that are intended for insertion into the vagina or the rectum) or the penetration of these items into the vaginal wall are also common occurrences [2, 3]. Insertion of an object into the vagina and its penetration of the vaginal wall will routinely result in that object penetrating into the abdominal cavity which leads to the possibility of a bowel injury [4, 5]. Penetration of an item into the rectum can lead to the perforation of the rectum or the possibility of the device becoming lodged so high in the rectum that it cannot be safely removed in the emergency room and requires operating room assistance. Penetration of a large object into the bladder and becoming entrapped in the bladder is rare.

We report a case of an unusually narrow vibrator becoming entrapped in the patient's bladder and mimicking the appearance of being intra-abdominal on physical exam and X-ray.

## 2. Case Report

**Figure 1:**
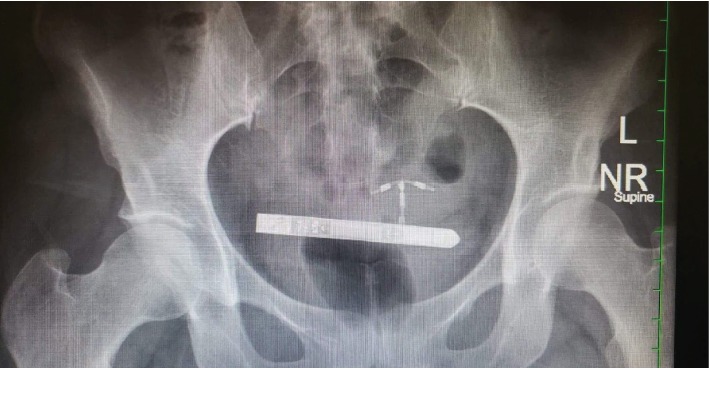
Abdominal and pelvic X-ray showing the device within the pelvis.

A 29-year-old gravida 1 para 0-0-1-0 Ab1 white female presented to the emergency room at approximately 1 a.m. after reporting that she lost her vibrator during sexual activity and could not find it. The patient remarked that she was using the vibrator for direct clitoral stimulation when her partner suddenly initiated vaginal intercourse. The patient remarked that she was uncertain of the location of the vibrator and felt some discomfort briefly, but believed that the vibrator was intravaginal as the intercourse took place. Following the vaginal intercourse, the patient was unable to find a vibrator in her vagina but still had the vibration sensation within her pelvis. When the patient was unable to find the vibrator, she presented to the emergency room. She remarked that the vibration in her pelvis lasted for approximately 30 minutes until stopping, presumably when the batteries lost all charge. A flat plate X-ray of the patient's pelvis showed the vibrator to be approximately at the level of the patient's intrauterine device in the pelvis. The vibrator was also in a horizontal position (Figure 1).

**Figure 2:**
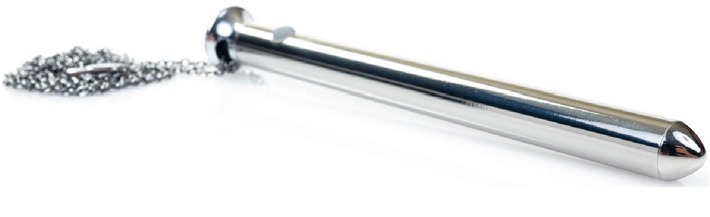
Vesper™ vibrator shown with removable chain attached.

Careful questioning of the patient showed that the vibrator was of an unusual type called a Vesper™, which is of unusually narrow diameter at approximately 1.2 cm (Figure 2).

**Figure 3:**
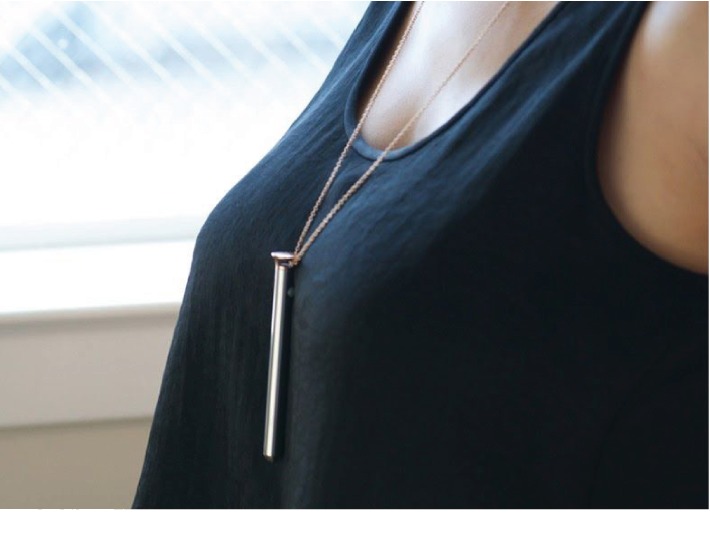
Vesper™ vibrator shown being worn as a necklace.

The device is approximately 10 cm long and is designed to be worn on a chain around the neck as a necklace to maximize the convenience of vibrator usage (Figure 3). The necklace is detachable in order to use the vibrator for sexual activities. Further questioning reveals that the patient had removed the chain necklace from the device before using it.

**Figure 4:**
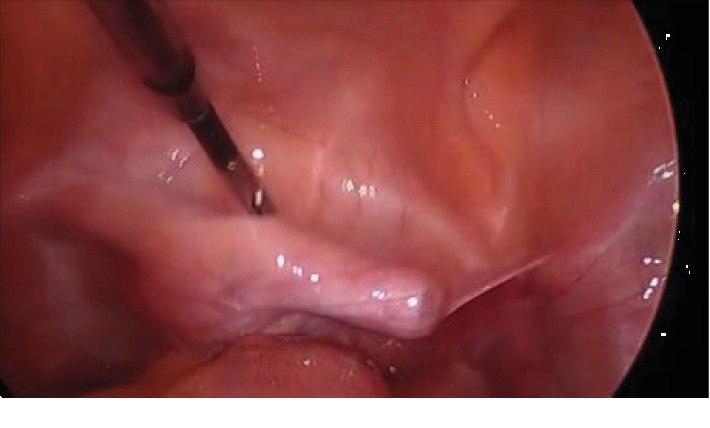
Laparoscopic view of the anterior cul-de-sac with foreign body in bladder.

Repeated vaginal and rectal examinations by the emergency room staff and the gynecologist showed no evidence of the device intravaginally or intrarectally, and the assumption was made that the device must have penetrated the vaginal wall and into the abdominal cavity. The patient had limited tenderness on examination; therefore, it was not assumed that rupture of the colon had occurred. However, the patient consented for possible colostomy and repair of the bowels prior to laparoscopic exploration. Laparoscopic exploration showed that the device was within the bladder, and this was demonstrated by gentle manipulation of the Foley bulb as well as by moving the sponge stick placed in the patient's vagina at the time of laparoscopy (Figure 4).

**Figure 5:**
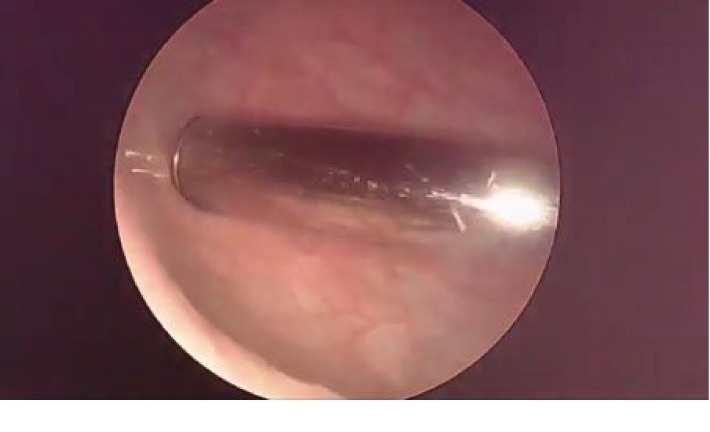
Device visualized in the bladder at the time of cystoscopy.

The vibrator was visualized at the time of cystoscopy (Figure 5) and was able to be removed with a cystoscopic grasper.

It was necessary to briefly fill the bladder with approximately one liter of normal saline in order to change the orientation of the vibrator from the horizontal to the longitudinal plane to remove it through the urethra safely. This did not result in any morbidity.

The patient did well and was a candidate for discharge immediately following removal of the foreign body.

## 3. Discussion

To provide the highest level of care and treatment of a foreign body, it is necessary to have the best understanding of the offending object possible [6–8]. Most gynecologists are intimately familiar with sex-related injuries and the related possible morbidities. One assumption that most gynecologic surgeons would generally make when assessing a situation such as this is that any standard vibrator would be of too great of a caliper in order to fit into the patient's urethra [9, 10]. In this case however, this uniquely narrow vibrator was later measured to be approximately 1.2 cm in total diameter, which is approximately the same diameter of a 36 French catheter [11, 12]. Therefore, when the device became lodged in the patient's urethra, sexual activity pushed the device into the bladder and the device became lodged in the bladder without puncturing it [13, 14].

Another interesting facet is the assumption that the device was in the pelvic cavity because it appeared so on X-ray. This fooled both the radiologist interpreting the study as well as the surgeon. From a gynecologic perspective, many surgeons have experience treating the aftermath of failed abortions were nonmedical practitioners have penetrated the posterior vaginal wall and penetrated the abdominal cavity. As a result of this assumption, laparoscopy was undertaken as the first course of action to retrieve the object, whereas cystoscopy was ultimately required. Knowledge of the anatomy was also required; there is a possibility of confusing placement in the bladder with the possibility that there would be some anatomical space between the vagina and the abdominal cavity in which the object could lodge, which is not the case.

Consideration could be given to counseling women to avoid devices with an extremely small diameter because of the possibility of urethral insertion.

## 4. Conclusion

An unusual vibrator with an extremely narrow diameter became lodged in the patient's bladder. The device was removed with cystoscopic manipulation following laparoscopic exploration. Although the patient did well and had no complications, laparoscopy could have been avoided if the possibility of intraurethral insertion had been considered preoperatively.
